# Preparation and characterization of amnion hydrogel and its synergistic effect with adipose derived stem cells towards IL1β activated chondrocytes

**DOI:** 10.1038/s41598-020-75921-w

**Published:** 2020-10-30

**Authors:** Maumita Bhattacharjee, Jorge L. Escobar Ivirico, Ho-Man Kan, Rosalie Bordett, Rishikesh Pandey, Takayoshi Otsuka, Lakshmi S. Nair, Cato T. Laurencin

**Affiliations:** 1 Connecticut Convergence Institute for Translation in Regenerative Engineering, University of Connecticut Health, 263 Farmington Ave, Farmington, CT 06030 USA; 2 Raymond and Beverly Sackler Center for Biomedical, Biological, Physical and Engineering Sciences, University of Connecticut Health, Farmington, CT USA; 3 Department of Orthopaedic Surgery, University of Connecticut Health, Farmington, CT USA; 4 Department of Chemical and Biomolecular Engineering, University of Connecticut, Storrs, CT USA; 5 Connecticut Children’s Innovation Center, School of Medicine, University of Connecticut Health, Farmington, CT USA; 6 Department of Biomedical Engineering, University of Connecticut, Storrs, CT USA; 7 Department of Materials Science and Engineering, University of Connecticut, Storrs, CT USA; 8 Institute of Materials Science, University of Connecticut, Storrs, CT USA; 9 Department of Craniofacial Sciences, School of Dental Medicine, University of Connecticut Health, Farmington, CT USA

**Keywords:** Mesenchymal stem cells, Diseases, Materials science

## Abstract

Inflammation leads to chondrocyte senescence and cartilage degeneration, resulting in osteoarthritis (OA). Adipose‐derived stem cells (ADSCs) exert paracrine effects protecting chondrocytes from degenerative changes. However, the lack of optimum delivery systems for ADSCs limits its use in the clinic. The use of extracellular matrix based injectable hydrogels has gained increased attention due to their unique properties. In the present study, we developed hydrogels from amnion tissue as a delivery system for ADSCs. We investigated the potential of amnion hydrogel to maintain ADSC functions, the synergistic effect of AM with ADSC in preventing the catabolic responses of inflammation in stimulated chondrocytes. We also investigated the role of Wnt/β-catenin signaling pathway in IL-1β induced inflammation in chondrocytes and the ability of AM-ADSC to inhibit Wnt/β-catenin signaling. Our results showed that AM hydrogels supported cell viability, proliferation, and stemness. ADSCs, AM hydrogels and AM-ADSCs inhibited the catabolic responses of IL-1β and inhibited the Wnt/β-catenin signaling pathway, indicating possible involvement of Wnt/β-catenin signaling pathways in IL-1β induced inflammation. The results also showed that the synergistic effect of AM-ADSCs was more pronounced in preventing catabolic responses in activated chondrocytes. In conclusion, we showed that AM hydrogels can be used as a potential carrier for ADSCs, and can be developed as a potential therapeutic agent for treating OA.

## Introduction

The articular cartilage homeostasis is maintained by chondrocytes, which regulate its structure, function as well as extracellular matrix (ECM) turnover. Abnormal conditions such as mechanical stress, inflammation, or accumulation of altered matrix proteins and degradation products, results in chondrocyte activation leading to disease conditions such as osteoarthritis (OA)^1^. Inflammation caused by the inflammatory mediators (tumor necrosis alpha (TNFα) and interleukin 1 beta (IL-1β) play a major role in the pathophysiology of cartilage damage and degradation in OA^2,3^. Chondrocytes treated with IL-1β release inflammatory mediators, which changes chondrocyte functions and promotes the expression of inflammation-related catabolic genes which results in further breakdown of cartilage ECM^4,5^.

Recently, the use of adipose-derived stem cells (ADSCs) has been widely explored to prevent inflammation and slow down the process of articular cartilage degeneration. ADSCs secrete a large number of anti-inflammatory and chondro- protective agents, which inhibit inflammation, suppress immune recognition and reduces apoptosis and the dedifferentiation of chondrocytes^6–8^. Our previous study demonstrated the potential of the paracrine effect of ADSCs in enhancing the anti-inflammatory effect in IL-1β activated ATDC5 cells^9^. Currently, stem cell therapy has certain challenges such as the lack of ideal cell carriers to maintain cell viability and localize the delivered cells at the target site for a required period. Thus, more effective cell delivery methods capable of sustaining the survival of implanted cells while maintaining their functions is needed to fully use the potential of cell therapy^10^. Considering these factors, promising new strategies have been developed using bio-mimetic and tissue-specific injectable hydrogels derived from various decellularized tissues such as heart, lungs, bones, etc. for stem cell delivery applications^11^. However, the efficacy of these hydrogels for stem cell delivery, while maintaining their viability and stemness and presenting their paracrine effects to regulate inflammation in osteoarthritic chondrocytes, has not been investigated.

An easily accessible ECM is the amnion membrane (AM) obtained from placental tissue. AM is the innermost layer of placental tissue and includes collagens (types I, III, IV, V and VI), fibronectin, laminin, proteoglycans, and hyaluronan. AM has been shown to suppress the expression of potent pro-inflammatory cytokines, such as IL-1α and IL-1β, and decrease matrix metalloproteinase (MMP) levels through the expression of natural MMP inhibitors present in the membrane^12^. AM also contains IL-1Ra, a receptor antagonist for IL-1, a pro-inflammatory cytokine that is upregulated in OA. We thus developed an amnion membrane (AM) hydrogel with the hypothesis that the AM hydrogel would retain ADSCs; maintain their viability, proliferation, and stemness, further modulating the catabolic responses (inflammation, expression of matrix degrading enzymes) in activated chondrocytes synergistically with ADSCs.

The present study, therefore, aimed to develop an injectable AM based hydrogel for ADSC delivery, which may together prevent the catabolic responses in IL-1β activated chondrocytes. Firstly, we designed and characterized AM hydrogels. Next, we evaluated the role of AM hydrogels in supporting ADSC survival, growth, proliferation and stemness. We evaluated the anti-inflammatory and chondro- protective effects of ADSCs and AM hydrogels on stimulated chondrocytes. We finally investigated the role of Wnt/β-catenin signaling which play a major role in transducing inflammatory and catabolic signals in joint degeneration.

## Results

### Decellularization and biochemical characterization of AM tissue

**Figure 1:**
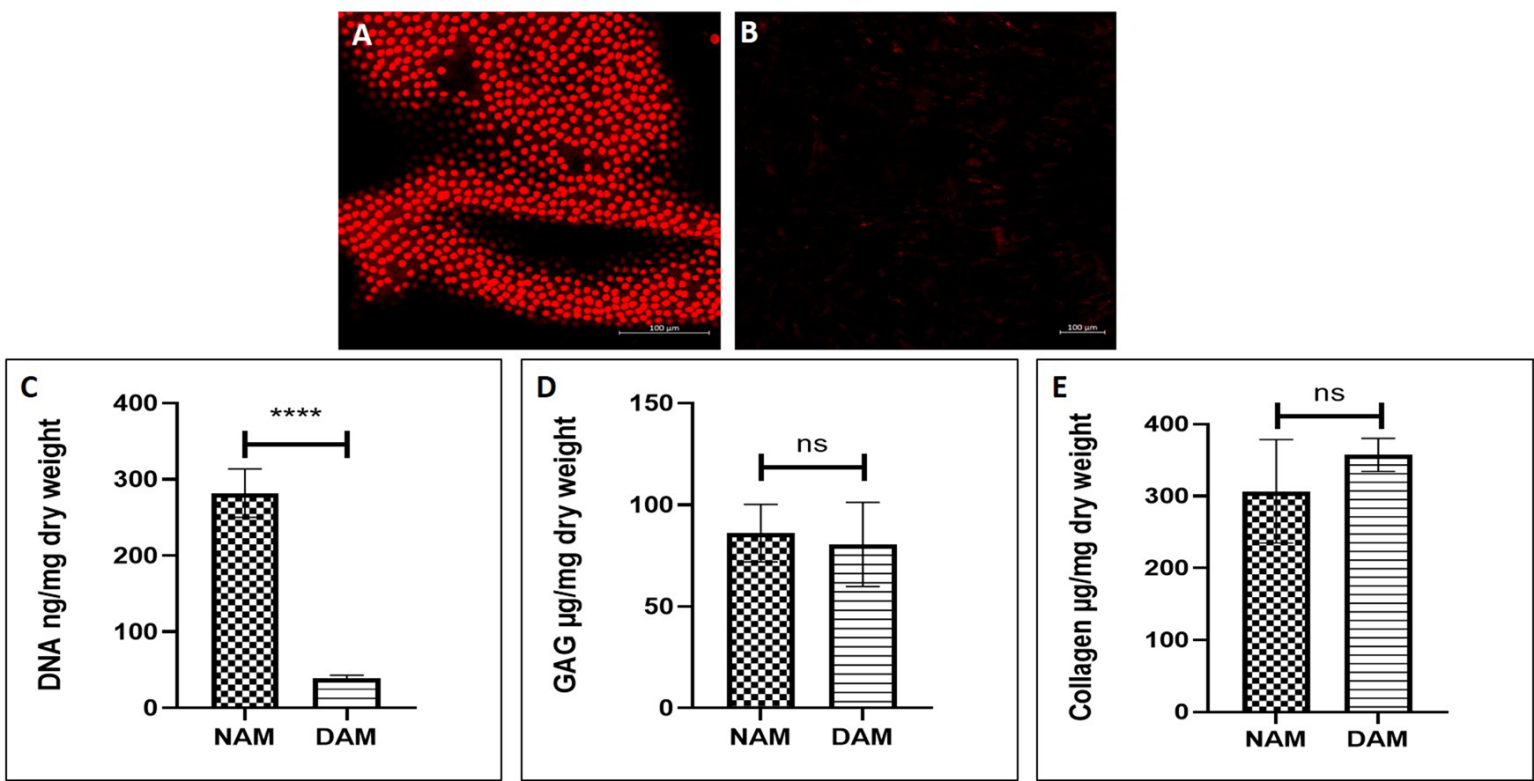
Decellularization of AM. (**A**) Native AM membrane, (**B**) decellularized AM membrane (scale bar 100 µm). (**C**) DNA quantification shows a significant reduction of DNA content in the decellularized AM (n = 6, ****p < 0.0001). (**D**) No significant difference between GAG content of native and decellularized AM (n = 8) and (**E**) No significant difference between total collagen content of native and decellularized AM, (n = 8).

In this study, we decellularized AM tissue using a simple alkaline lysis method and the decellularization process was evaluated by PI staining to detect residual DNA on the AM tissue. The control group (AM tissue without decellularization) showed substantial amounts of DNA, indicating the presence of AM epithelial cells (Fig. 1A). In contrast, PI staining of decellularized AM tissue samples showed no detectable DNA residues (Fig. 1B). The results from PI staining on decellularization was then confirmed by quantifying the DNA content of both native and decellularized AM using a PicoGreen assay. Quantifying DNA content by measuring double stranded DNA (dsDNA) is considered a potential indicator of remaining cell debris and a quantitative extent of decellularization. The native AM tissue showed 281.0 ± 31.8 ng of DNA per mg dry weight in comparison to decellularized AM, which contained only 38.8 ± 4.2 ng of DNA per mg dry weight, strengthening our decellularization data (Fig. 1C). To evaluate whether the decellularization process affected the matrix components we quantified the glycosaminoglycan and total collagen content in both native and decellularized AM. The native AM contained 86.09 ± 14.1 µg GAGs per mg of dry tissue weight whereas the decellularized AM showed 80.4 ± 20.6 µg GAGs per mg of dry tissue weight. There was no significant difference in the GAG content of the native and decellularized AM tissue, indicating that our decellularization treatment did not affect the GAG content (Fig. 1D). To determine the collagen preservation after decellularization hydroxyproline quantification assay was performed. The results showed that native AM contained 306 ± 71.9 µg hydroxyproline per mg of dry tissue weight and the decellularized AM showed 357 ± 22.9 µg hydroxyproline per mg of dry tissue weight (Fig. 1E). No significant difference was observed in the collagen content between the groups indicating no loss of collagen after the decellularization process.

### Design and characterization of AM hydrogels

**Figure 2:**
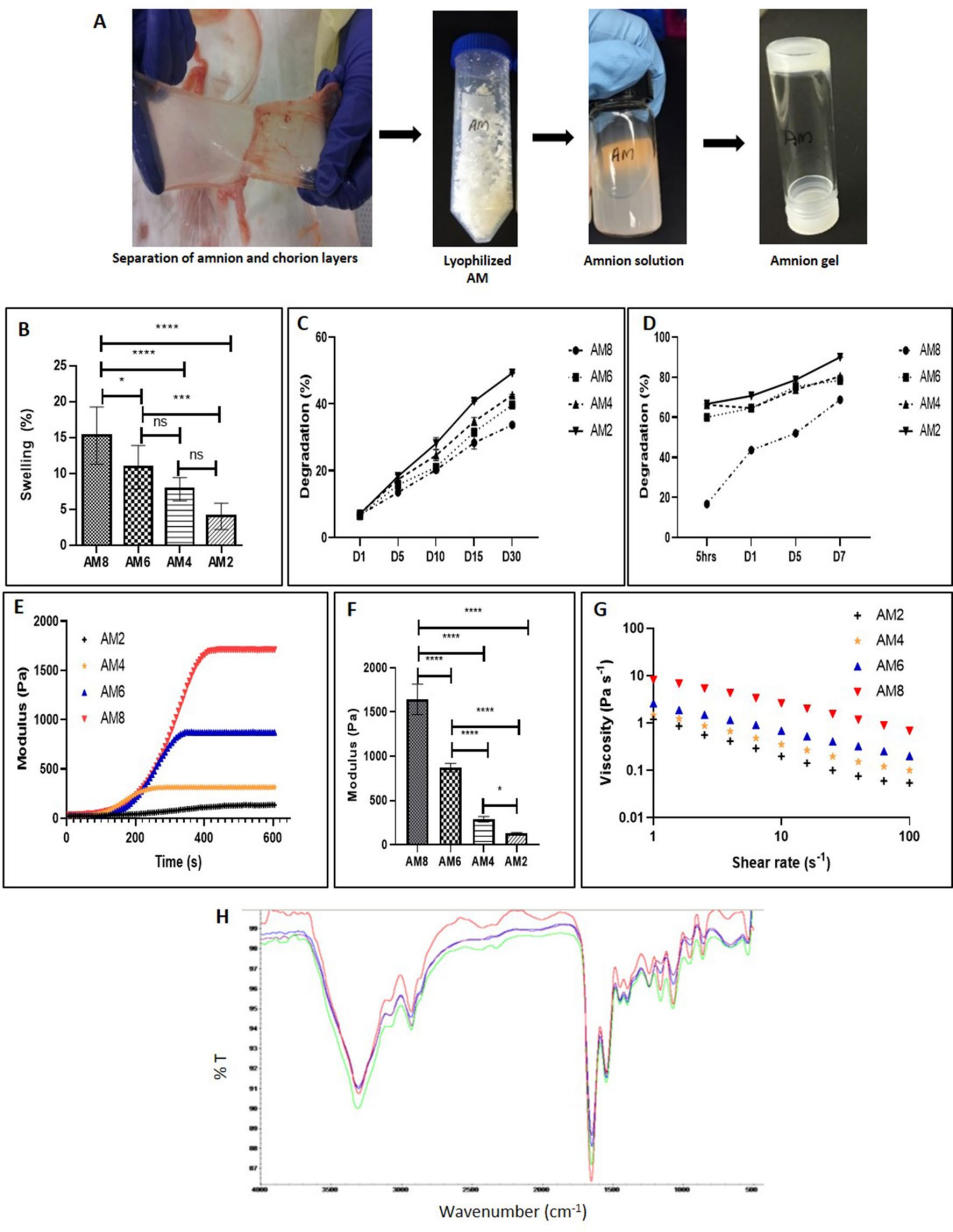
(**A**) Schematic representation of AM hydrogel preparation. Increasing AM concentration increases swelling and decreases dissolution and degradation. (**B**) % swelling of AM hydrogels after 24 h; n = 4 repeated twice showing mean and SD, [****p < 0.0001, ***p < 0.001, *p < 0.05]. (**C**) % dissolution of AM hydrogels in PBS; n = 3 showing mean and SD. (**D**) % degradation of AM hydrogels in collagenase I (10 units/mL); n = 3 repeated twice showing mean and SD, AM concentration increases modulus and viscosity. (**E**) Representative curves of the gelation of AM hydrogels; (**F**) Storage modulus of AM hydrogels; n = 3 repeated twice showing mean and SD (*p < 0.05, ****p < 0.0001). (**G**) Viscosity profile of AM hydrogels. (**H**) ATR-FTIR of AM hydrogels showing characteristic absorption bands of collagen. Figure (**A**) pictures taken by Maumita Bhattacharjee and Jorge L. Escobar Ivirico.

The preparation process of the AM hydrogel is presented in Fig. 2A. After decellularization, lyophilized AM was digested enzymatically to solubilize the AM. The soluble AM matrix is a free flowing liquid which self-assembled into a hydrogel when brought to physiological ionic strength, pH, and temperature of 37 °C for 5–10 min. AM gelation was mediated by self-assembling molecules such as collagens, laminins, and proteoglycans present within its matrix^13,14^. The hydrogels were successfully prepared from AM matrix at various concentrations: 8, 6, 4, and 2 mg/mL and were found to be injectable through a syringe with minimal resistance, an important parameter to be a clinically relevant injectable material for minimally invasive delivery. Lower concentration of AM hydrogels were prepared as use of higher concentration would clog the needle and would not facilitate proper cell delivery. Concentrated hydrogels (8 and 6 mg/mL) were found to be more rigid macroscopically, compared to the less concentrated hydrogels which were weaker and difficult to handle.

The swelling percentage of the AM hydrogels, 8, 6, 4, and 2 mg/mL was measured in PBS gravimetrically (Fig. 2B). The AM hydrogels showed a swelling percentage ranging from 5 to 15%. The degradation of AM hydrogels in different concentration was followed by 30 days in an enzyme free PBS media which showed moderate weight loss of AM gels and enzyme containing PBS media. The degradation rate increased with the decreasing concentration of the hydrogels (Fig. 2C). By 30 days AM 8 mg/mL showed only about 33% degradation, followed by AM 6 mg/mL (39%), 4 mg/mL (42%) and 2 mg/mL (49%). Degradation properties were also studied in PBS containing 0.035 mg/ml (10 U/ml) collagenase I (accelerated enzymatic degradation). The presence of enzymes significantly increased the degradation. Similar to the enzyme free degradation study, in the presence of enzyme lower concentration AM hydrogels showed faster degradation by 7 days (Fig. 2D). Degradation was found to be 90% in AM 2 mg/mL followed by 80% in AM 4 mg/mL, 78% in 6 mg/mL and 68% in 8 mg/mL after 7 days of treatment.

The storage modulus (G’) of AM hydrogels was found to change over time characterized by a sigmoidal curve shape after the temperature of the sample was raised from 10 to 37 °C as shown in Fig. 2E,F. The modulus increased with increasing AM concentration, thus increasing concentrations of AM hydrogels leads to increased stiffness. The modulus (Pa) were calculated to be 1643 Pa for AM 8 mg/mL, 871 Pa for AM 6 mg/mL, 287 Pa for AM 4 mg/mL, and 126 Pa for AM 2 mg/mL, respectively (Fig. 2E,F). The viscosity of the AM hydrogels with different concentrations was then measured over a range of shear rates, as viscosity is an important parameter while considering a material for injection. The AM hydrogels at different concentrations showed similar linear profiles when plotted on a log–log scale, with a decrease in the complex viscosity as the shear rate increased (Fig. 2G) indicating that they are shear thinning. Moreover, with the increase in AM hydrogel concentration there was an increase in viscosity as observed by the vertical shift of the data points.

ATR-FTIR spectral analysis (Fig. 2H) shows 3 characteristic absorption bands of collagenous tissue at 1652 cm^−1^ (amide I, C=O stretching), 1550 cm^−1^ (amide II, N–H bending), and 1339 cm^−1^ (amide III, N–H bending) in all the AM hydrogels. The spectra also exhibited peaks at 3315 and 2936 cm^−1^, assigned to the stretching vibrations of N–H and C–H bonds, respectively. The peaks at 1454 cm^−1^ signify the –C–O stretching present in the –O–COCH3 group and those observed at 1082 cm^−1^ correspond to the aliphatic chain with a primary amino group. The band at 1240 cm^−1^ is indicative of S=O stretching of R-SO3^–1^ characteristic of polysaccharides from 1200 to 1000 cm^−1^^15,16^.

### AM based hydrogel can support ADSC survival, proliferation and stemness

**Figure 3:**
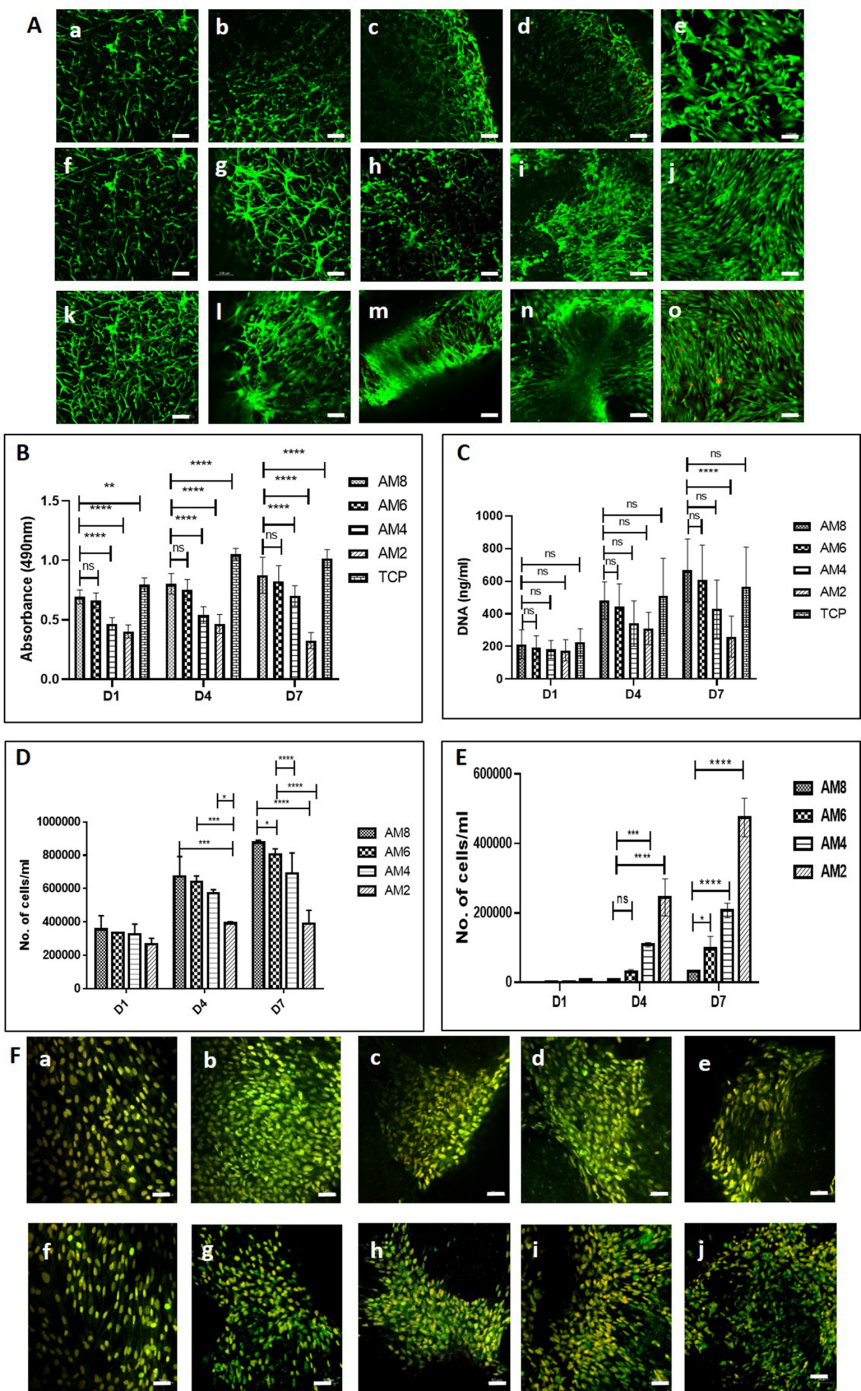
(**A**) AM concentration did not affect cell viability. Live dead assay: (a–e) Day 1—AM8, AM6, AM4, AM2, tissue culture plate (TCP; control); (f–i) Day 4—AM8, AM6, AM4, AM2, (TCP; k–o); Day 7—AM8, AM6, AM4, AM2, (TCP), respectively for each group, n = 3, repeated twice (scale bar 100 µm). Low AM concentration reduces cell proliferation and increases migration. (**B**) MTS assay of ADSCs in different AM hydrogels. (**C**) DNA quantification (**D**) Cell count by flow cytometry in AM hydrogels and (**E**) Cell count by flow cytometry in plate which migrated out of the hydrogels, D = day; mean and SD, n = 3, repeated twice [****p < 0.0001, ***p < 0.001, **p < 0.01, *p < 0.05]. (**F**) ADSCs proliferated on all AM hydrogel concentrations. Ki-67 staining showing cell proliferation; Day 4 (a–e), (a) TCP (b) AM8 (c) AM6 (d) AM4 (e) AM2 hydrogels, Day 7 (f–j), (f) TCP (g) AM8 (h) AM6 (i) AM4 (j) AM2 hydrogels, n = 3, repeated twice (scale bar 50 µm).

We then investigated whether the different concentrations of AM hydrogels can support ADSCs viability and proliferation. ADSCs were isolated from rat fat pads and characterized by flow cytometry (Supplementary data Fig. S1). The hydrogels at all concentrations showed the presence of viable cells as evaluated by LIVE/DEAD cell viability assay with a few dead cells after days 1, 4 and 7 indicating that the cell viability was maintained within the AM hydrogels (Fig. 3A). The adherence and spreading of ADSCs within the AM hydrogels is due to the presence of binding domains present in the AM. The ADSC proliferation was evaluated quantitatively by an MTS assay, DNA quantification, and flow cytometry and qualitatively by Ki-67 staining. Ki-67 protein, which is associated with cell proliferation, was found to be expressed by ADSCs encapsulated within all the hydrogels after 4 and 7 days of culture (Fig. 3F) as well as on TCP suggesting that cells cultured in hydrogel have the proliferative ability. The MTS assay revealed that all concentrations of AM hydrogels supported ADSC viability and proliferation. Cell proliferation was increased in AM 8, 6 and 4 mg/mL from day 1 to day 7. However, cell proliferation in AM 2 mg/mL was reduced on day 7 (Fig. 3B). Of the different concentrations used AM 8 mg/ml showed highest proliferation which indicated that concentration has an effect on cell proliferation. DNA quantification and flow cytometry analysis also revealed a similar pattern of cell proliferation as observed by the MTS assay. Cell proliferation increased from day 1 to day 7 in AM 8, 6 and 4 mg/mL but not in AM 2 mg/mL (Fig. 3C–E). This was due to shrinking of AM 2 mg/ml hydrogel after cell encapsulation and migration of cells from the hydrogel into the plate. To verify that the decrease in cell proliferation in lower concentration AM hydrogels is due to hydrogel shrinking, we counted the number of cells within the hydrogels (Fig. 3D) and the cells which migrated from the hydrogel to the plate by flow cytometry (Fig. 3E).

The flow cytometry data showed that cells migrated out of the hydrogel due to shrinking causing cell proliferation by MTS assay and DNA quantification to decrease in AM 4 and AM 2 mg/mL hydrogels (Fig. 3B,C). The results confirmed that the AM hydrogels irrespective of the concentration are biocompatible and can provide a suitable microenvironment for maintaining the ADSC viability and proliferation up to 7 days. Flow cytometry and gene expression analysis showed that the AM hydrogels maintained stemness of ADSC upto 7 days. (Supplementary data Figs. S2, S3).

### Evaluating the anti-inflammatory and chondro-protective effects of ADSC and AM based hydrogels and the involvement of Wnt/β-catenin signaling pathways

The experimental groups were divided into 5 groups- Group 1: chondrocyte alone (control), Group 2: chondrocytes with IL-1β (20 ng/mL), Group 3: chondrocytes with IL-1β (20 ng/mL) and ADSCs, Group 4: chondrocytes with IL-1β (20 ng/mL), and AM (6 mg/mL) and Group 5: chondrocytes with IL-1β (20 ng/mL) and ADSCs encapsulated within AM (6 mg/mL) hydrogel. AM 6 mg/ml was chosen as it can be injected easily and also maintains stem cells functions compared to other concentrations.

#### MTS assay and nitric oxide (NO) assay

**Figure 4:**
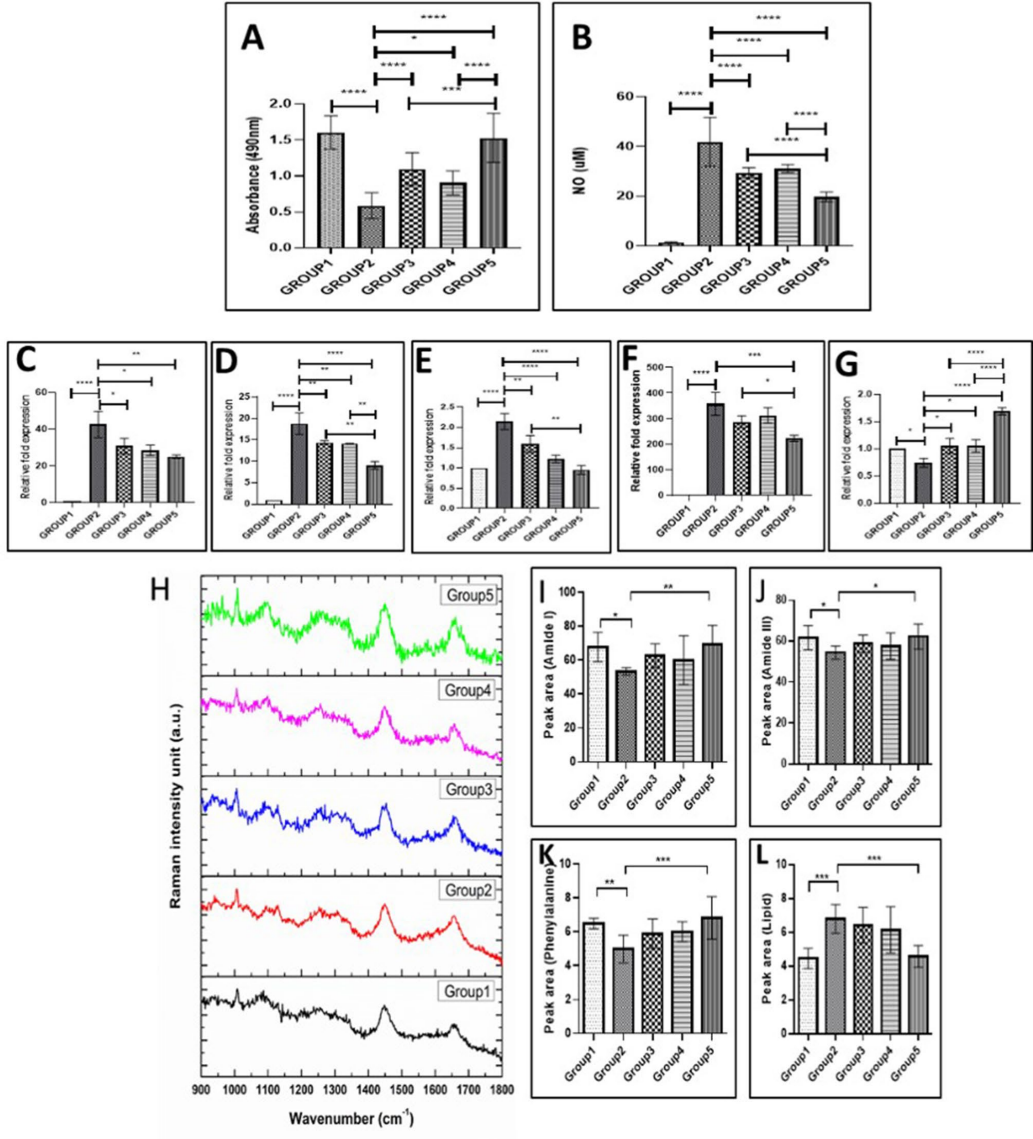
AM-ADSCs exhibited the highest cell viability and lowest NO production. (**A**) MTS assay for cell viability and (**B**) NO assay for NO production, mean and SD with n = 4, repeated thrice [****p < 0.0001, ***p < 0.001, *p < 0.05]. ADSCs-AM exhibited reduced catabolic and cytokine and increased TIMP gene expression. Gene expression analysis of (**C**) MMP-3, (**D**) MMP-13, (**E**) ADAMTS5, (**F**) IL-6, and (**G**) TIMP-1, mean and SD with n = 4, repeated twice [****p < 0.0001, ***p < 0.001, **p < 0.01, *p < 0.05]. (**H**) Normalized Raman spectra of rat chondrocytes with different treatments. Group 1: untreated chondrocytes, Group 2: chondrocytes + 20 ng/mL IL-1β, Group 3: chondrocytes + IL-1β (20 ng/mL) + ADSCs, Group 4: chondrocytes + IL-1β (20 ng/mL) + AM (6 mg/ml), and Group 5: chondrocytes with IL-1β (20 ng/mL) and ADSCs encapsulated within AM. Peak area quantification of amide-I (**I**), amide III (**J**), phenyl alanine (**K**), lipid (**L**), mean and SD with n = 3, repeated thrice [***p < 0.001, **p < 0.01, *p < 0.05].

MTS assay showed that the cell viability was found to reduce upon IL-1β (20 ng/mL) treatment compared to the control group. The addition of ADSCs (Group 3), AM hydrogel (Group 4) and AM-ADSCs (Group 5) was found to significantly increase the cell viability of chondrocytes treated with IL-1β (20 ng/mL). In addition, Group 5 (AM-ADSCs) showed the highest cell viability indicating the synergistic chondro-protective effect of the AM hydrogel and ADSC in preventing the effect of pro-inflammatory cytokines (Fig. 4A). IL-1β (20 ng/mL) treatment increased NO production in Group 2 compared to the control group. The treatment with ADSCs (Group 3), AM hydrogel (Group 4) and AM-ADSCs (Group 5) was found to significantly reduce the NO production in chondrocytes treated with IL-1β (20 ng/mL). Similar to the MTS assay, we observed that Group 5 (AM-ADSCs) showed the lowest NO production compared to Groups 3 (ADSCs) and 4 (AM hydrogel), confirming the synergistic effect of AM-ADSC (Fig. 4B).

#### Gene expression analysis to evaluate the effect of ADSCs and AM hydrogels on cytokine treated chondrocytes

To investigate the inhibition of catabolic responses of IL-1β by ADSCs, AM hydrogels and AM-ADSCs, RT-PCR was performed to determine the gene expression level of inflammatory marker and matrix-degrading enzymes. The IL-1β-treated group induced chondrocyte catabolism by increasing the mRNA expression levels of the matrix-degrading enzymes MMP-3, MMP-13, ADAMTS5, and the pro-inflammatory cytokine IL-6, compared to those in the untreated control group. The treatment groups, Group 3 (ADSCs), Group 4 (AM hydrogel) and Group 5 (AM-ADSCs) were found to attenuate the increased mRNA level of IL-6, MMP-3, MMP-13, and ADAMTS-5 in chondrocytes caused by IL-1β. The expression of TIMP was found to reduce upon treatment with IL-1β. However, the treatment groups, Group 3 (ADSCs), Group 4 (AM hydrogel) and Group 5 (AM-ADSCs) increased TIMP expression in IL-1β-treated cells (Fig. 4C–G). Similar to MTS and NO results, Group 5 (AM-ADSCs) showed significant downregulation of IL-6, MMP-3, MMP-13, and ADAMTS-5 and upregulation of TIMP compared to other groups indicating synergistic chondro-protective and anti-inflammatory effect of the AM hydrogel and ADSC in preventing the catabolic effects of pro-inflammatory cytokines.

#### Raman spectroscopic analysis to evaluate the effect of ADSCs and AM hydrogels on cytokine treated chondrocytes

We performed Raman microscopic investigation of chondrocytes from groups 1–5. The normalized mean Raman spectra of chondrocytes with different treatment groups acquired in the identical conditions are shown in Fig. 4H. As evident from the figure, chondrocytes of the different treatment groups display subtle differences in their spectral patterns. The spectra were analyzed by quantifying the peak area of prominent Raman bands namely amide-I (1657 cm^−1^), amide-III (1267 cm^−1^), phenylalanine (1004 cm^−1^) and lipid (1304 cm^−1^). As evident from Fig. 4I, the amide I peak area decreased significantly in IL-1β treated chondrocytes (Group 2) as compared to the untreated control group (Group 1). Further, the area under the amide I peak increased, albeit insignificant, in Group 3 and 4, where IL-1β treated chondrocytes were subjected to ADSCs and AM hydrogels, respectively. On the other hand, Group 5 (AM-ADSC) showed a statistically significant increase in the amide I area compared to Group 2 reversing the effect of IL-1β. We observed similar trends for other protein bands such as amide III (1267 cm^−1^) and phenylalanine (1004 cm^−1^) (Fig. 4 J,K). Interestingly, the area of the 1304 cm^−1^ band attributed to lipids, increased significantly in Group 2 and reversed in Group 3 and 4. Notably, we observed a substantial reduction in the lipid peak area in Group 5 (Fig. 4L) showing a synergistic effect of AM-ADSC in protecting chondrocytes.

Together, the MTS assay, NO assay, gene expression studies and Raman spectroscopic studies showed statistically significant difference in Group 5 compared to Group 3 and 4. Thus we conclude that synergistic effect of amnion and ADSC (Group 5) can better inhibit the catabolic responses in IL1β activated chondrocytes.

#### Role of Wnt/β-catenin signaling in modulating the inflammatory response

**Figure 5:**
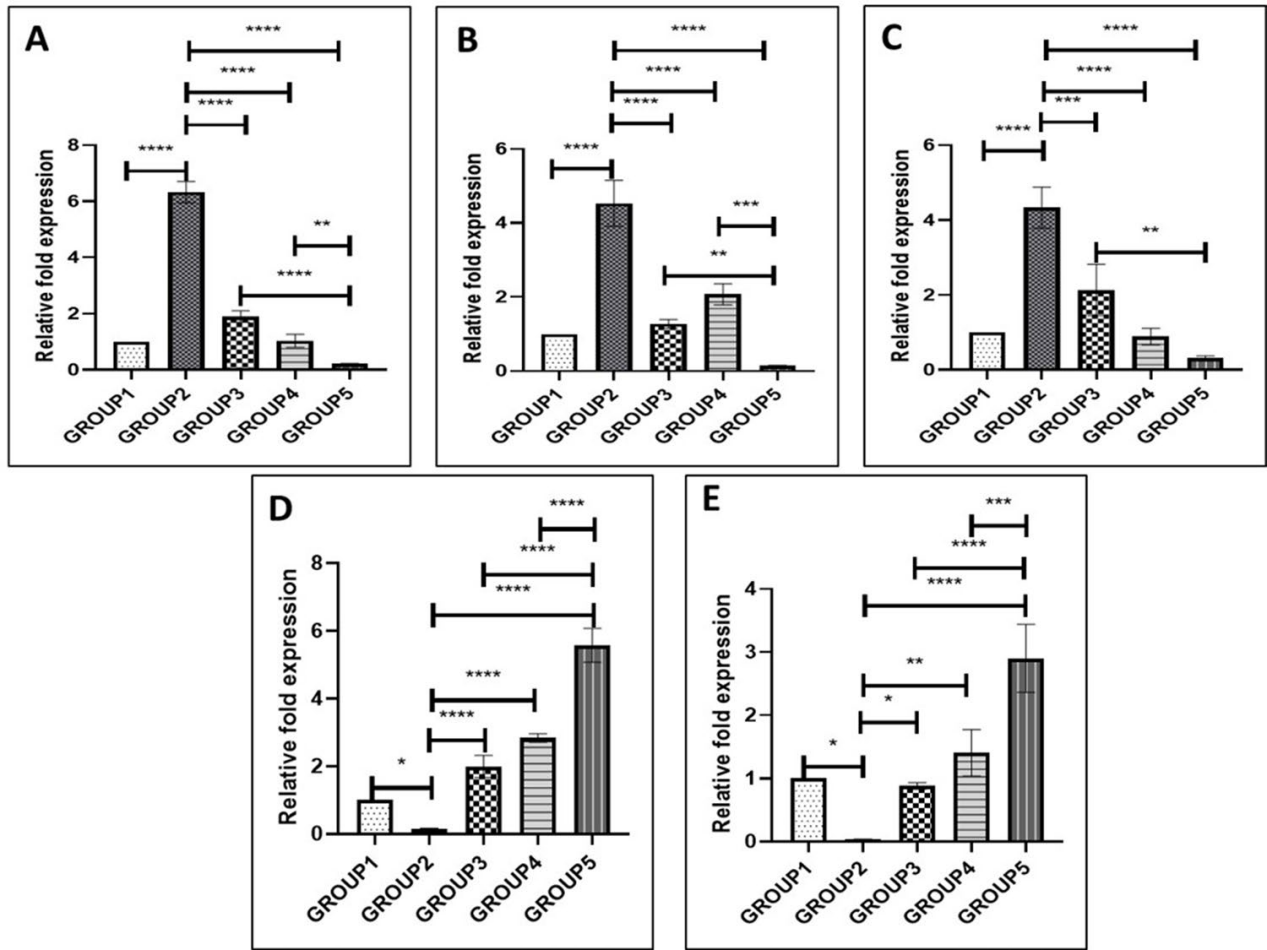
Involvement of Wnt/β-catenin signaling pathways in chondrocyte inflammation (group 2) and ADSC, AM hydrogel and AM-ADSCs downregulating the Wnt/β-catenin signaling pathway members: (**A**) LRP5, (**B**) LRP6 and (**C**) β-catenin, mean and SD with n = 3, repeated twice [***p < 0.001, **p < 0.01]. (**D**,**E**) Gene expression showing Wnt/β-catenin antagonist DKK1 and FRZB downregulated in group 2 (IL-1β treated chondrocytes) and ADSCs, AM hydrogel and AM-ADSCs group showed upregulation of DKK1 and FRZB genes, with significantly higher expression in the AM-ADSCs group thus inactivate Wnt signaling pathways, mean and SD with n = 3 [****p < 0.0001, ***p < 0.001, **p < 0.01, *p < 0.05].

We investigated the involvement of Wnt/β-catenin signaling pathway in IL-1β induced inflammation in chondrocytes. Further, we investigated the ability of ADSCs, AM hydrogel and AM-ADSCs as inhibitors of Wnt/β-catenin signaling to block IL-1β induced chondrocyte activation. As shown in Fig. 5, the expression of LRP5/6 and β-catenin was upregulated in IL-1β-stimulated rat chondrocytes. This suggests that the Wnt/β‑catenin signaling pathway was activated and may be involved in chondrocyte inflammation. Treatment with ADSC, AM hydrogel and AM-ADSCs downregulated the expression of Wnt/β-catenin signaling pathway members LRP5/6 and β-catenin (Fig. 5). The inhibition of the Wnt/β-catenin signaling pathway was significantly higher in the combination group of AM-ADSCs.

We finally investigated the expression of Wnt antagonists, Dickkopf-1 (DKK1) and Frizzled related protein (FRZB). As shown in Fig. 5 D and E, IL-1β treatment of healthy chondrocytes caused a significant reduction in mRNA levels of DKK1 and FRZB. Whereas, the treatment groups, ADSC, AM hydrogel and AM-ADSCs showed upregulation of DKK1 and FRZB genes, with significantly higher expression in the AM-ADSC group.

## Discussion

Decellularized tissues are widely used for biomedical applications. However, complete decellularization of tissue is important for minimizing potentially negative immune responses, host acceptance, and improve tissue remodeling outcomes^17^. Previously, different decellularization methods have been used such as dispase, trypsin, SDS, EDTA, and mechanical scraping with an electric toothbrush. These methods are tedious, long and have adverse effects on tissue architecture and ECM composition^18,19^. To overcome these problems we decellularized AM by gently rubbing a cotton swab soaked in NaOH over the AM which led to nearly complete and easy removal of adherent cells without using harsh chemicals and techniques. The DNA content in the decellularized AM was also below the threshold of 50 ng/mg of dry ECM^18,19^ (Fig. 1C) which may be an advantage to avoid detrimental immune response following injection.

Preservation of matrix components is key to provide an optimum environment with appropriate biological and chemical cues important for cell–matrix interactions essential for cell migration, proliferation, differentiation, mechanical stability, and signaling. Previous methods on decellularization have reported a severe loss of matrix components with 50% collagen loss and 80% sulfated GAG loss^20,21^ resulting in severe matrix damage, which would impair cell engraftment due to the loss of fibronectin and complete loss of matrix strength due to collagen loss, rendering a depleted matrix not suitable for tissue regenerative applications. It was observed that the optimized protocol in our study prevented the loss of matrix components and retained collagen and GAG which is similar to the native tissue (Fig. 1D,E). Thus, our decellularized AM can be used as a potential biomaterial for cell delivery and tissue regeneration applications without evoking any immunological reaction, paving the way toward achieving successful tissue regeneration.

AM hydrogels may have several desirable features for therapeutic applications including targeted delivery of ADSCs by minimally invasive techniques, ease of repeated delivery, ability to quickly fill an irregularly shaped space, and polymerization to form a support structure suitable for host cell infiltration and remodeling. Injectable AM hydrogels, thus, have the potential advantage of syringe/catheter delivery and by in situ gelation can provide structural support for cell localization within the target site. The increase in swelling percentage with the increasing concentration of the hydrogel (Fig, 2B) could be due to the presence of polar molecules such as sGAG which increases the water uptake^22^. AM hydrogels were found to degrade in a concentration dependent manner (Fig. 2C,D). Accelerated degradation study using 35 μg/ml of collagenase was performed to understand the AM degradation profile within a short duration of time. However, we believe that AM gel will show slower degradation in vivo due to low collagenase content^23^. Also the potential of AM gels to elevate the levels of TIMP, which would inhibit collagenase, could further slowdown the degradation rate of the AM gel. It is important to study the degradation behavior of these hydrogels as degradation induces structural or conformational alteration in the hydrogels. Such alterations would reveal the biologically active cryptic sites within matrix molecules (matricryptic sites) involved in controlling cell shape, migration, proliferation, differentiation, morphogenesis, and survival^24^. The rapid and complete degradation of the AM hydrogels indicates that they have the potential to promote and facilitate proper tissue growth and remodeling via the release of matricryptic molecules.

AM hydrogels were found to undergo fast gelation as shown by rheology studies (Fig. 2E), which is possibly due to the presence of self-assembling components and the molecular interaction between collagen and other components of the AM matrix. Previous studies have shown that the extracellular matrices are a macromolecular assembly of natural biopolymers including collagens, GAGs, proteoglycans, and glycoproteins, which can undergo gelation by a process called self-directed polymerization, "self-assembly". GAGs act as a nucleation site for collagen fibrillogenesis and proteoglycans are known as regulator of fibril self-assembly. In addition, other matrix proteins such as fibronectin is also known to organize collagen fibers, and other minor collagens as nucleation sites for collagen fibrillogenesis^25,26^. The presence of collagen, GAGs, proteoglycans, fibronectin in AM possibly facilitates faster gelation. Faster AM hydrogel gelation is desired for in vivo and clinical applications to limit the cell loss from the site of application. Also, the AM hydrogels showed concentration dependent increased viscosity and shear thinning property. The increase in viscosity with increasing concentration of hydrogel is attributed to the increased intermolecular forces leading to an increase in viscosity^27^. Shear-thinning property is another very important factor for the translation of an injectable hydrogel as highly viscous or shear thickening material may block the syringe/ catheter. The sol-hydrogel transition at 37 °C and shear thinning properties of AM hydrogels would allow for direct injection and gelation in situ at body temperature into a tissue specific location.

In vitro cell characterization revealed that AM hydrogels supported ADSCs functions such as viability, proliferation, and stemness (Fig. 3 and supplementary data Figs. S2, S3). The increased cell proliferation with the increasing AM concentration which could be attributed to the stiffness of the hydrogels. AM 8 mg/mL showed the highest cell proliferation as it was the stiffest compared to the other AM hydrogels. Similar outcomes have been shown in previous studies where bone marrow derived stem cells and fibroblast proliferation has been shown to increase with increased hydrogel stiffness^28,29^. Also, the reason for the decrease in proliferation from high to low concentration of AM hydrogel can be attributed to the shrinking/contraction of the hydrogels caused by the contractile forces generated by the ADSCs. Shrinking of hydrogels results in maximum cell migrating out of the hydrogel to the plate. Shrinking/contraction is a well-defined phenomenon observed in collagen and ECM hydrogels seeded with fibroblast/ MSCs that are known to generate tension on the matrix during both extension and retraction of pseudopodia^30^.

The paracrine effect of ADSCs is known to play an important role in exerting a protective effect in a local tissue environment. Thus, we investigated the paracrine effect of ADSCs within the hydrogel and synergistic role of AM-ADSCs to inhibit the catabolic responses of inflamed chondrocytes using a transwell system. In this study, the inflammatory condition was induced by treating rat chondrocytes with IL-1β, a condition close to the inflammatory environment occurring in cartilage during OA. Several lines of evidence have confirmed the involvement of IL-1β and its catabolic responses in cartilage degradation and OA inflammation^31,32^. Thus, IL-1β was chosen as a pro-inflammatory mediator to induce inflammation in the chondrocytes. Our findings (Fig. 4A) are in agreement with previous studies, which showed that ADSCs induces autophagy in IL‐1β stimulated chondrocytes by promoting the expression of autophagy related genes (LC3-II and p63). Autophagic activation attenuates chondrocyte apoptosis and reduces MMPs and ADAMTSs expression in chondrocytes, thereby controlling inflammation^32^. As chondrocyte apoptosis plays an important role in OA development and progression the increased survival of chondrocytes by ADSC, AM hydrogel and AM-ADSCs might be a protective mechanism induced by the treatment groups. During the inflammatory process, Nitric Oxide (NO) is generated by activated iNOS which inhibits the synthesis of collagen and proteoglycans and increases MMP activity. IL-1β (20 ng/mL) treatment increased NO production in Group 2 similar to previous studies which showed IL-1β induces chondrocytes to release inflammatory mediators such as NO, prostaglandin E2 (PEG2), proteolytic enzymes ADAMTS and MMPs which further breakdown the matrix components, resulting in the alteration of the biochemical composition of chondrocytes^33^. Interestingly, the treatment groups reversed the effect of IL-1β and Group 5 (AM-ADSCs) showed the highest cell viability and lowest NO indicating a synergistic effect of the AM hydrogel and ADSC in preventing the effect of pro-inflammatory cytokines.

ADSCs are known to reduce the expression of inflammatory factors and expression of matrix degrading factors by secreting multiple immunosuppressive factors, like IL-10, IL-1 Receptor Antagonist (IL-1RA), TGF-β and induce anti-inflammatory effects in macrophages. Moreover, AM also exhibited potent anti-inflammatory effects by inhibiting the expression of pro-inflammatory cytokines such as IL-1α and IL-1β, and decreasing MMP levels^12^. It was reported that AM produced compounds having both antimicrobial and anti-inflammatory properties, including β-defensins, secretory leukocyte proteinase inhibitor (SLPI) and elafin^34,35^. These unique properties of AM and ADSCs could be attributed to the synergistic chondro-protective and anti-inflammatory properties evidenced in our study.

Apart from various biochemical assays, Raman spectroscopy (RS) was used to detect the bio-molecular changes at a single cell resolution in inflamed rat chondrocytes upon treatment with pro-inflammatory cytokine IL-1β and whether these changes can be reversed by treating them with ADSCs, AM hydrogel and AM-ADSCs^36,37^. RS has also been employed to study chondrocytes during the progression of OA based on biochemical compositions at the cellular level^38^. However, the application of RS to systematically investigate the anti-inflammatory effects of AM-ADSCs on IL-1 β treated chondrocytes has not been previously studied. Our RS observations indicated a general decrease in protein content in the IL-1β treated chondrocytes, which were reversed significantly upon the treatment with the AM-ADSC group (Fig. 4H–K). We reasoned that the Raman spectral pattern of the cell would undergo discernible change(s) after the treatment with pro-inflammatory cytokine IL-1β and the spectral change would report on the biomolecular alteration. Kumar et al.^38^ investigated chondrocytes isolated from different stages (ICRS Grade-I, II and III) of osteoarthritic cartilage, and noted a decrease in the area of protein peaks (amide I, amide III and Phenylalanine) with osteoarthritic progression. The increased lipid deposition has been associated with osteoarthritis progression and advancements^39–42^ and the inflammation leads to an increase in the deposition of lipid droplets. Czamara et al.^37^ noted that incubation of endothelial HMEC-1 cells with the pro-inflammatory cytokine TNFα resulted in increased accumulation of lipid bodies. Lipid bodies contain a lipid core surrounded by a monolayer of phospholipids with associated proteins^41,43^ and are responsible for the production of various inflammatory mediators in response to different inflammatory factors^43^. Further, Kumar et al. also noted that Raman intensity of the lipid band 1304 cm^−1^ increases with the progression of osteoarthritis^34^ Our results displayed a similar outcome where the area corresponding to the lipid peak significantly increased in response to inflammation caused by IL-1β treatment, and interestingly, this lipid deposition was subsequently reduced by the synergistic effect of AM-ADSCs (Fig. 4L). Altogether, RS investigation corroborated our MTS, NO, and RT-PCR data confirming the synergistic anti-inflammatory and chondro-protective effect of AM-ADSC, which inhibited the catabolic responses in activated chondrocytes. Thus, the combination of ADSCs with AM may hold tremendous potential in treating inflammation and cartilage degradation in OA patients.

Wnt/β-catenin signaling pathway has been previously shown to contribute to the ECM damage process and chondrocyte destruction in the etiology and pathogenesis of OA^44^. During the Wnt/β-catenin signaling process, the extracellular Wnt ligands bind to receptors (Frizzled) and co-receptors (LRP 5/6) which then facilitate translocation of key protein β-catenin into the nucleus and activating it. Activated β-catenin in articular chondrocytes has been shown to induce hypertrophy and expression of ECM degradation proteins, and matrix mineralization leading to an OA like phenotype^45^. Our result showed the involvement of Wnt/β‑catenin signaling pathway in IL-1β activated chondrocytes (Figs. 4, 5A–C). This is in agreement with previous reports where IL-1β has been shown to increase LRP’s and β–catenin in chondrocytes^46^. LRP receptors play very important role in transducing Wnt signaling and inducing nuclear β-catenin localization in cartilage. LRP5 has been shown to be upregulated in OA and LRP6 has been associated with an increased OA progression^47,48^. Because Wnt/β-catenin signaling was found to be involved we hypothesized that inhibition of Wnt/β-catenin signaling might be an effective therapeutic strategy for the treatment of cytokine-induced chondrocyte inflammation and cartilage degradation. Interestingly, treatment groups were found to inhibit the Wnt/β-catenin signaling pathway (Figs. 4, 5A–C). A similar finding was reported in our previous work where ADSCs inhibited Wnt/β-catenin signaling in ATDC5 cells treated with IL-1β^9^. However, the Wnt/β-catenin signaling inhibition was found to be more pronounced in the AM-ADSCs group indicating the synergistic effect of AM and ADSCs.

Further, to establish the crosstalk between IL-1β and Wnt/β-catenin signaling in chondrocyte inflammation, we investigated whether IL-1β upregulates Wnt/β-catenin signaling via downregulating Wnt antagonists, Dickkopf-1 (DKK1) and Frizzled related protein (FRZB). A previous study by Zhong et al.^45^ showed that IL-1β upregulates Wnt/β-catenin signaling pathways via downregulating Wnt antagonists, Dickkopf-1 (DKK1) and Frizzled related protein (FRZB). The inhibition of Wnt antagonist in IL-1β treated group (Fig. 5D,E) would activate the Wnt/β-catenin signaling pathway and lead to chondrocyte inflammation and degradation, whereas the upregulation of Wnt/β-catenin antagonist in the treatment groups (Fig. 5D,E) would inactivate Wnt/β-catenin signaling pathways, preventing chondrocyte inflammation and degradation. It has been proved that human chondrocytes treated with IL-1β showed reduced expression of DKK1 and FRZB through upregulation of nitric oxide synthase (iNOS) which synthesizes NO, thereby, activating the transcription of Wnt/β-catenin target genes^45^. Our study showed similar results, wherein, stimulated chondrocytes showed increased NO production and reduced DKK1 and FRZB expression. Thus, from our findings and in co-relation with previous studies, we can highlight that IL-1β played a role in initiating chondrocyte inflammation and destruction by increasing Wnt/β-catenin activity via NO production and by reducing DKK1 and FRZB expression in rat chondrocytes. Further, the reduction of NO and upregulation of Wnt/β-catenin antagonist, DKK1 and FRZB expression in treatment groups showed the ability of our treatment groups in inhibiting Wnt/β-catenin signaling pathways. Interestingly, the combination of AM and ADSCs showed significant upregulation of DKK1 and FRZB, showing the maximum effect on Wnt/β-catenin signaling pathway inhibition. However, further studies are needed to elucidate the exact mechanism and factors involved in determining the chondro-protective and anti-inflammatory effects of AM and ADSCs to provide additional mechanistic insights.

In conclusion, we demonstrated the feasibility of developing an AM-based injectable hydrogel from the placental tissue. The properties of AM hydrogels were tuned by altering the AM concentration. The efficacy of AM hydrogels to support ADSC viability, proliferation, and stemness was demonstrated. Our studies also showed that ADSCs and AM could synergistically exert anti-inflammatory and chondro-protective ques by targeting catabolic and inflammatory mediators. The effect of ADSCs and AM on IL-1β-induced chondrocytes might be associated with the inhibition of the Wnt/β-catenin signaling pathway showing potential for OA treatment. Considering the unique features of AM and the properties of injectable AM hydrogel, it can be used as a promising carrier for ADSCs to prevent chondrocyte inflammation and cartilage breakdown during OA. However, the effect and mechanism of action of AM and ADSCs should be further explored in an in vivo environment and clinical situations, as the present study was limited to in vitro studies.

## Methods

### AM Tissue collection and decellularization

Discarded, de-identified placental tissue was collected after getting approval from the institutional ethical committee (University of Connecticut, Health, USA). Additionally, the isolation methods were performed in accordance with the experimental guidelines and regulations approved by Institutional Review Board (IRB), University of Connecticut, Health, USA (Study number-IE-08-310-1). AM and chorion layers were mechanically delaminated from the fresh placental tissue. Under sterile conditions harvested AM was washed with normal saline containing 100 units/mL of penicillin and 100 μg/mL of streptomycin to remove blood and mucus. Next, the membrane was decellularized using 0.5 M NaOH followed by two cycles of rinsing the membrane with sterile deionized (DI) water. Decellularized AM was then lyophilized and stored at room temperature. The decellularization process was verified by propidium iodide (PI) staining for DNA which examines for the absence of nuclei. Briefly, AM tissue sections were fixed in acetone, rehydrated, rinsed in water, stained for 10 min (min), rinsed with PBS and imaged using confocal microscope (Zeiss LSM 880 confocal Microscope).

### Biochemical quantification

The biochemical composition (DNA, glycosaminoglycan (GAG), and its collagen contents) of native and decellularized AM were quantified using various biochemical quantification methods. The tissues were lyophilized and 1 mg of dry AM tissue was digested with 3 units/mL papain at 60˚C before analysis. The measured values were normalized to the dry weight of the samples.

#### DNA quantification

DNA was isolated and quantified using a Quant-iT PicoGreen dsDNA assay kit (Thermofisher Scientific) following the manufacturer’s instructions. Briefly, after papain digest, DNA samples were mixed with the Quant-iT PicoGreen reagent and the samples were excited at 480 nm and the fluorescence emission intensity was measured at 520 nm via spectrofluorometer. The DNA content was quantified using a standard curve and normalized to the dry AM tissue weight.

#### Glycosaminoglycan quantification

To directly measure the sulfated glycosaminoglycan (GAG) content, papain-digested samples were stained with 1,9-dimethylmethylene blue (DMB, Sigma) and photometrically measured at 525 nm as described. A dilution series of chondroitin sulfate in PBS was used as the standard solution^49^. The GAG content was normalized to the dry weight of the AM tissue.

#### Collagen quantification

The samples were dried and analyzed using a hydroxyproline assay kit (Sigma) and hydroxyproline as a standard^50,51^. The collagen content was normalized to the dry weight of the AM tissue.

### AM hydrogel preparation and characterization

#### Preparation of AM hydrogels

To develop an injectable form of AM, the decellularized AM was first lyophilized, ground into a coarse powder and solubilized enzymatically. The AM was digested in a porcine pepsin solution in a ratio of 10:1 (AM: Pepsin). Pepsin was used at a concentration of 1 mg/mL using 0.01 N HCl. The mixture was then stirred at room temperature for 48 h. The AM digests were neutralized to a pH of 7.4 by adding 0.1 M NaOH, followed by 10× PBS. The neutralized AM was then diluted to the desired final AM concentration (8, 6, 4 and 2 mg/mL) with 1X PBS on ice. The AM pre-hydrogels were then placed in an incubator heated to 37 °C to form hydrogels.

#### Swelling analysis of AM hydrogels

Swelling percentages of the AM hydrogels (8, 6, 4, and 2 mg/mL) were measured gravimetrically following methods from a previous study^52^. After gelation, the samples (n = 4 per group) were incubated in PBS (pH = 7.4) at 37 °C for 24 h, in order to measure their wet weight at maximum saturation. The swelling percentage of different hydrogels was calculated using the following equation: swelling *(%)* = *(W*_*w*_ − *W*_*i*_*)*/*W*_*w*_ × *100*, where Ww and Wi are the weights of the hydrogels in the equilibrium swelling state and initial gelling state, respectively.

#### Degradation of AM hydrogels

The degradation of AM hydrogels (8, 6, 4, and 2 mg/mL) was studied in two types of degradation solutions, 1 mL of PBS (pH 7.4) with or without collagenase (10 units/mL, Invitrogen) as described previously 50. The AM hydrogels (8, 6, 4, and 2 mg/mL) were equally weighed, immersed in 10 mL degradation media, and continuously oscillated at 37 °C. Hydrogels in PBS were removed after 0, 1, 5, 10, 15, and 30 days, whereas hydrogels in the collagenase-containing medium were collected after 5 h, 1, 5 and 7 days. Finally, all the collected hydrogels were freeze-dried and weighed (*W*_t_). The dry weight of the hydrogels obtained at day 0 was noted as the initial weight (*W*_i_). The degradation of the hydrogel was calculated using the following formula:

$$ {\text{Degradation }}\% \, = \, W_{{\text{i}}} {-} \, W_{{\text{t}}} /W_{{\text{i}}} \, \times \, 100 $$

#### Rheological measurements

The rheological measurements of the AM hydrogels (8, 6, 4, and 2 mg/mL) were carried out by Discovery HR3 hybrid Rheometer (TA Instruments), using 12 mm parallel plate geometry. The sample (100 µL) was injected using a 16 G needle onto the rheometer plate by filling its gap (gap width: 980 mm). An oscillatory time sweep was carried out to measure the gelation of the forming AM hydrogel over time at 37 °C (a temperature at which AM gelation occurs) and applying 1% oscillatory strain at a frequency of 1 Hz. The complex viscosity ǀ η^*^ǀ of AM hydrogels was determined by performing a frequency sweep between 0.01 and 10 Hz at 37 °C and 10% strain.

#### Attenuated total reflection-Fourier transform infrared spectroscopy (ATR-FTIR)

ATR-FTIR spectroscopy was performed to confirm the presence of characteristic AM bands. Different concentrations of AM hydrogels were prepared, frozen and lyophilized. Infrared spectra for AM hydrogels were measured by ATR-FTIR in the spectral range of 4000–500 cm^−1^ using an accumulation of 264 scans with a resolution of 4 cm^−1^. A background scan was obtained in the absence of material, and the baseline was normalized for each sample after the acquisition.

### In vitro cell culture experiments

#### Adipose derived stem cell (ADSCs) isolation and characterization

ADSCs were isolated from 6–8 week-old Sprague Dawley (SD) rats around 250 g as reported in ur previous study^53^ in accordance with the experimental guidelines and regulations approved by University of Connecticut Health Center Institutional Animal Care and Use Committee (IACUC) approved protocol. Additionally, ADSC isolation was done following the institutional IACUC approved protocol. Rats were euthanized with CO_2_ inhalation followed by neck dislocation. Euthanized rats were weighed, shaved and cleaned with 70% Ethanol. Inguinal fat pads were isolated and weighed. The fat pads were collected and washed thrice in sterile HBSS with 1% Anti-anti followed by mincing the tissues into small pieces. An equal volume of collagenase type I (0.1%) (Invitrogen) in HBSS was added to the fat tissue and agitated at 37 °C for 90 min. The cell suspension was filtered through a 100 μm filter (BD Bioscience) for the removal of the solid aggregates. The collagenase was neutralized by adding an equal volume of DMEM-F12 with 10% FBS and 1% Anti-anti. The mixture was centrifuged at 1500 rpm for 10 min and 2 mL of red cell lysis buffer was added to the pellet and incubated for 2 min. The mixture was centrifuged again at 1500 rpm for 10 min. The supernatant was discarded and cells were counted and plated in a T25/T75 flask containing DMEM-F12 with 10% FBS and 1% Anti-anti. The media was changed after 24 h. Next, the cells were cultured for 2 weeks, according to standard procedures, in DMEM-F12 supplemented with 1% penicillin/streptomycin. ADSCs reaching 80–90% confluence were detached with 0.25% trypsin (Invitrogen) at 37 °C for approximately 5 min, centrifuged at 1500 rpm for 10 min and re-plated. ADSCs were characterized by flow cytometry using cell surface markers (Becton–Dickinson LSR II, BD Biosciences, USA) at passage 3 (P3). Cells were washed using sterile PBS, centrifuged and re-suspended in sterile FACS buffer (PBS, 1% FBS) containing 10 μL of the FITC-conjugated CD29 antibody, FITC conjugated CD90 antibody, FITC conjugated CD105, FITC conjugated CD45, and PE conjugated CD11b for 30 min. Unlabeled cells were used as controls. Cells were then scanned with FACS; acquired and gated using forward scatter (FSC) and side scatter (SSC) parameters to exclude cell debris and aggregates.

#### ADSC encapsulation with AM hydrogels

The ADSCs were encapsulated in different concentrations of AM hydrogels by the following method. The cells were trypsinized using 0.25% trypsin–EDTA at P3. The isolated ADSCs were mixed with different concentrations of AM solution to form cell-hydrogel constructs containing 1 × 10^6^ cells per mL of the pre-hydrogel solution. The cell suspension mixed with the pre-hydrogel solution was slowly dropped into well plates and allowed to form a gel in an incubator at 37 °C and 5% CO_2_. After gelation (about 10 min for all concentrations) the wells were filled with DMEM-F12 containing 10% FBS, 1% pen/strep for up to 7 days. Cells were seeded on tissue culture plate (TCP) and considered as the control group.

#### ADSC viability and proliferation

The LIVE/DEAD cell viability assay was used to visualize the distribution of living and dead cells in the hydrogel at different time points. The hydrogels were washed in PBS and incubated with 4 mM calcein-AM and 2 mM ethidium homodimer-I at 37 °C. Fluorescence images were taken using confocal microscope (Zeiss LSM 880 Confocal Microscope). The cell proliferation with AM hydrogels and TCP was detected by Ki-67 immunostaining, a proliferation marker. The hydrogels were washed with PBS followed by fixing in cold 100% Methanol for 5 min, permeabilized with 0.5% Triton‐X for 30 min followed by incubation with blocking buffer (2% bovine serum albumin) for 1 h. The hydrogels were incubated with the primary antibody for Ki-67 (Invitrogen, 1:100 dilution) for 1 h followed by incubation with the secondary antibody for Ki-67 (Alexa Fluor 488 goat anti‐rabbit, Invitrogen, 1:200 dilution). The hydrogels were then stained with PI for 30 min and imaged using confocal microscope (Zeiss LSM 880 confocal Microscope).

The metabolic activity, viability and proliferation of cells in the hydrogels were assessed using the CellTiter 96 Aqueous nonradioactive cell proliferation assay (MTS assay; Promega) following the manufacturer's protocol. Briefly, the hydrogels were collected in a new plate and washed with PBS. MTS reagent in a ratio of 5:1 (media: MTS) was added to each well at each time point. The plates were then incubated for 4 h at 37 °C and 5% CO_2_. The absorbance of the resulting solution was read at 490 nm using a microplate reader.

Cell proliferation was detected by using PicoGreen fluorescent DNA quantification (Molecular Probes) kit at days 1, 4 and 7 for both AM hydrogels and TCP. The hydrogels were washed with PBS and digested with collagenase I (0.1%) at 37 °C for 60 min. DNA samples were mixed with the Quant-iT PicoGreen reagent, measured via spectrophotometry at 520 nm with excitation at 485 nm and compared with a DNA standard curve provided. The cell proliferation within the hydrogels and plate was further confirmed by flow cytometry. The cells were isolated from the hydrogels and plate, suspended in PBS and counted using MACSQUANT analyzer (Miltenyi Biotech).

#### ADSC stemness within AM hydrogels

ADSCs at P3 were encapsulated within the different concentrations of AM hydrogels as previously described. After days 1, 4 and 7 the hydrogels were collected in a new plate and washed with PBS. For flow cytometry analysis the hydrogels were digested with collagenase I (0.1%) for 60 min at 37 °C, followed by washing the cell pellet in PBS. Cells were stained with 10 μL of the FITC conjugated CD29 antibody, FITC conjugated CD90 antibody, FITC conjugated CD105, FITC conjugated CD45, PE conjugated CD11b, FITC conjugated CD31, and PE conjugated CD34 for 30 min. Unlabeled cells were used as controls. Cells were then scanned with FACS, acquired and gated using forward scatter (FSC) and side scatter (SSC) parameters to exclude cell debris and aggregates.

Quantitative real-time PCR was employed to determine the ADSC stemness within the hydrogels and TCP. Total RNA was isolated using the RNeasy Mini Kit (Qiagen) according to the manufacturer's instructions. For cDNA synthesis, 2 μg total RNA was used as a template for Sprint RT Complete cDNA synthesis kit (Clontech) in a total volume of 20 μL. For quantitative real-time PCR, iCycler Thermal Cycler Base (Bio-Rad) and iQ Supermix (Bio-Rad), Sox-2, Oct-4 and GAPDH gene probes were used. The threshold cycle values of target genes were standardized against GAPDH expression and normalized to the expression in the control culture. The fold change in expression was calculated using the ΔΔCt comparative threshold cycle method^53^.

### Anti-inflammatory and immunosuppressive effects of ADSC and AM hydrogels

#### In vitro primary chondrocyte culture

Rat chondrocytes were obtained from Articular Engineering. The primary culture of chondrocytes was performed using rat articular chondrocytes which were plated in tissue culture flasks at a density of 10,000–20,000 cells per cm^2^. The chondrocytes were maintained in the chondrocyte growth medium containing 10% serum and 5 μg/mL gentamicin. When cells were subconfluent, they were detached by sequential treatment with 0.25% trypsin–EDTA.

#### Co-culture of ADSCs and chondrocytes

To investigate the paracrine effect of ADSCs on chondrocytes, we used a co-culture system following methods from our previous study^9^. Chondrocytes were plated into 24-well plates at a density of 1 × 10^5^ /well and cultured for 24 h. The culture medium was then refreshed with DMEM-F12 containing 20 ng/mL IL-1β to induce chondrocyte inflammatory responses for a further 24 h in the experimental group (Group 2). IL-1β treated chondrocytes were co-cultured with transwell inserts (Corning) containing 1 × 10^5^/well ADSCs (Group 3), 100 µL AM hydrogel (6 mg/mL) (Group 4) and 100 µL AM hydrogel (6 mg/mL) with 1 × 10^5^ ADSCs (Group 5). The cells were cultured in 2 mL DMEM-F12 supplemented with 5% FBS, 1% pen/strep and 20 ng/mL IL-1β. Chondrocytes without IL-1β served as the negative control (Group 1). Chondrocytes treated with 20 ng/mL IL-1β served as the positive control (Group 2). The chondrocytes were tested by MTS, NO assay kit, and RT-PCR. The concentration of NO was measured by the Griess reagent (Thermofisher Scientific) according to the manufacturer’s instruction. The media from each group was collected and treated with Griess reagent in room temperature for 30 min. Samples were then measured in a microplate reader at 570 nm. Gene expression studies were done as described above with the following primers: MMP3, MMP13, ADAMTS-5, IL-6, and TIMP-1.

#### Raman spectroscopy

Rat chondrocytes were cultured on quartz coverslips (~ 0.25 mm thick, Tedpella, Inc) in a 24-well plate. Co-culture methods as described above were used for the Raman spectroscopic studies. After culturing for 72 h, the chondrocytes on quartz coverslips were placed on a secure-seal spacer (Invitrogen, Thermofisher Scientific, Waltham) and subsequently sealed using another quartz glass.

Raman measurements were performed at 785 nm excitation in the backscattering configuration using a free-space custom-built Raman microspectroscopy system as described earlier^55^. Briefly, the Raman microscope consists of a 193 mm focal length spectrograph (Shamrock 193i, Andor, Belfast, UK) equipped with a thermoelectric cooled CCD camera (iDus DU420A-BEX2-DD, Andor). The power at the sample was held constant at 25 mW and Raman spectra were acquired from individual live single cells with a total of 10 cells from each group. The integration time for a single Raman measurement was 30 s and 2 accumulations were averaged. Both excitation and collection were performed using the same 60× objective with the numerical aperture of 1.1 (LUMFLN60XW, Olympus, Tokyo, Japan). Raman spectra were preprocessed by removing cosmic rays, subtracting background and smoothing. The preprocessed spectra were then subjected to peak normalization (0, 1) and the areas of key peaks such as amide-I (1657 cm^−1^), amide-III (1267 cm^−1^), phenylalanine (1004 cm^−1^) and lipid (1304 cm^−1^)^35^ were calculated using Origin Pro 8 (OriginLab Corp., USA) software program.

#### Wnt/β-catenin signaling analysis

To investigate the involvement of Wnt/β-catenin signaling pathways, the co-culture method described above was used and the members of the Wnt/β-catenin signaling pathways were analyzed by RT-PCR using predesigned LRP5, LRP6 and β-catenin primers (Thermofisher Scientific) following methods described above. The expression of Wnt/β-catenin antagonist was also analyzed using RT-PCR using predesigned primers, DKK1 and FRZB (Thermofisher Scientific).

### Statistical analysis

Student's t‐test of two samples assuming equal variance and ANOVA single factor tests were used to compare data between samples. Only significant differences were included in the images to facilitate the interpretation of the data.

## Supplementary information

Supplementary information
