# Supplementary material for: Preparation and characterization of amnion hydrogel and its synergistic effect with adipose derived stem cells towards IL1β activated chondrocytes
Source: Sci Rep. 2020 Oct 30;10:18751. doi: 10.1038/s41598-020-75921-w (PMC7603317; doi:10.1038/s41598-020-75921-w)
Supplement: Supplementary file 1 — Supplementary information [file 41598_2020_75921_MOESM1_ESM.docx]

**Preparation and characterization of amnion hydrogel and its synergistic effect with adipose derived stem cells towards IL1β activated chondrocytes**

Maumita Bhattacharjee^1,2,3^, Jorge L. Escobar Ivirico^2,3,4^, Ho-Man Kan^1,2,3^, Rosalie Bordett^5^, Rishikesh Pandey^5^, Takayoshi Otsuka^1,2,3^, Lakshmi S. Nair^1,2,3,6,7,8^, Cato T. Laurencin^1,2,3,4,6,7,8,9*^

*^1^ Connecticut Convergence Institute for Translation in Regenerative Engineering, University of Connecticut Health, Farmington, CT, USA*

*^2^Raymond and Beverly Sackler Center for Biomedical, Biological, Physical and Engineering Sciences, University of Connecticut Health, Farmington, CT, USA*

*^3^Department of Orthopaedic Surgery, University of Connecticut Health, Farmington, CT, USA*

*^4^Department of Chemical and Biomolecular Engineering, University of Connecticut, Storrs, CT, USA*

*^5^Connecticut Children's Innovation Center, School of Medicine, University of Connecticut Health, Farmington, CT, USA*

*^6^Department of Biomedical Engineering, University of Connecticut, Storrs, CT, USA*

*^7^Department of Materials Science and Engineering, University of Connecticut, Storrs, CT, USA*

*^8^Institute of Materials Science, University of Connecticut, Storrs, CT, USA*

*^9^Department of Craniofacial Sciences, School of Dental Medicine, University of Connecticut Health, Farmington, CT, USA*

* Corresponding author:

**Cato T. Laurencin M.D., Ph. D.**

University Professor

Albert and Wilda Van Dusen Distinguished Professor of Orthopaedic Surgery

Professor of Chemical, Materials and Biomolecular Engineering

Director, Connecticut Convergence Institute for Translation in Regenerative Engineering, University of Connecticut Health, Farmington, CT, USA

Director, The Raymond and Beverly Sackler Center for Biomedical, Biological, Physical and Engineering Sciences

The University of Connecticut

263 Farmington Ave, 06030, Farmington, CT.

E-mail: [Laurencin@uchc.edu](mailto:Laurencin@uchc.edu)

**1. ADSC characterization and encapsulation within AM hydrogels**


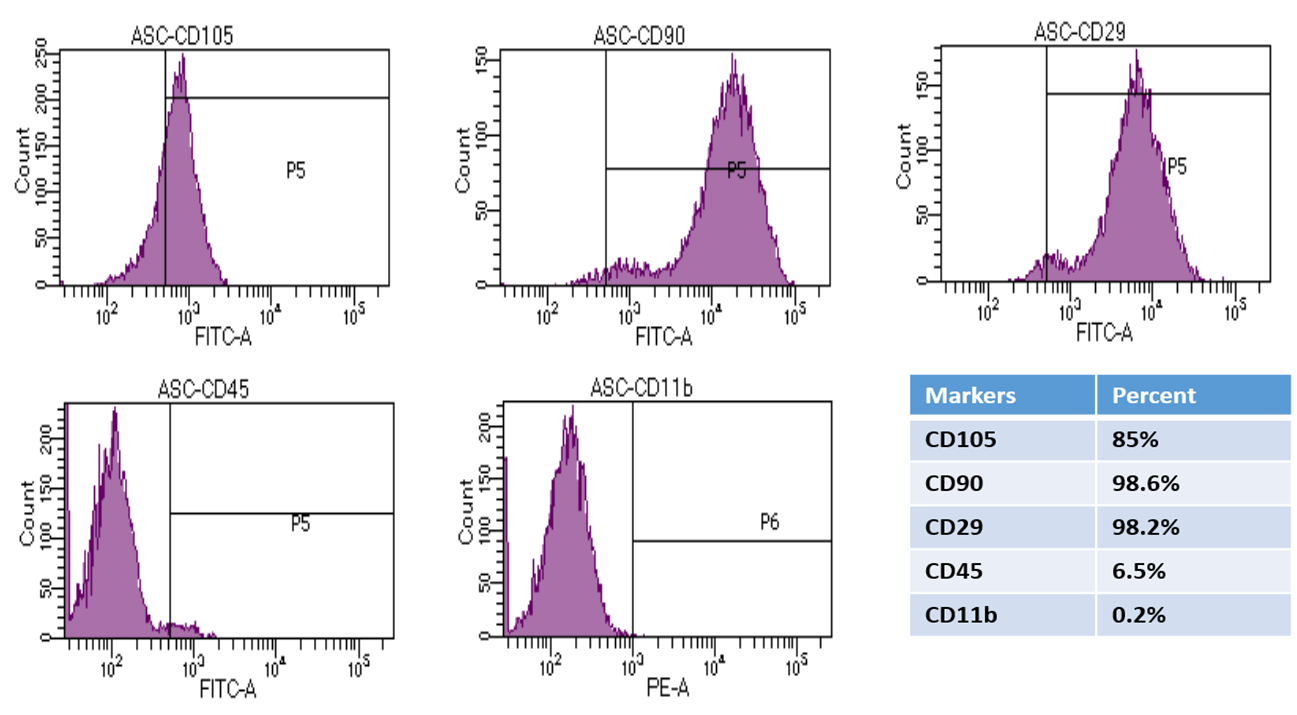
Adipose-derived stem cells were successfully isolated from rat inguinal fat pads. After 1 day of culture, spindle-shaped cells were found to be attached to the T flasks. The primary ADSC culture took 5-6 days to reach confluence. ADSCs at P3 were analyzed for expression of mesenchymal stem cell (MSC) specific cell-surface markers. ADSCs were found to be negative for the hematopoietic lineage marker CD45 (6.5%), and negative for CD11b (0.2%). ADSCs were found to be positive for CD29 (98.2%), CD90 (98.6%) and CD105 (85%). These results are consistent with previously published studies. ADSCs (P3) at a concentration of 1 × 10^6^ cells/mL were encapsulated within different concentrations of AM hydrogels 8, 6, 4 and 2 mg/mL and cultured for up to 7 days in DMEM-F12 containing 10% FBS, 1% pen/strep. The cells within the hydrogels were characterized on days 1, 4 and 7.

Fig S1. Flow cytometry analysis of rat ADSCs (P3)

**2. AM based hydrogel can support ADSC stemness**

It is believed that the therapeutic properties of MSCs are derivative of their paracrine activity thus, retention of cell stemness is a key factor for the secretion of trophic factors needed for tissue regeneration. Thus, to investigate the changes in the ADSC characteristics within the AM hydrogels we performed flow cytometry and gene expression analysis. Flow cytometry analysis revealed the presence of positive and absence of negative CD markers on ADSCs encapsulated within the AM hydrogels. The ADSCs within all the AM hydrogels were found to be positive for stem cell markers CD90 and CD29 after days 1, 4, and 7. Moreover, they were found to be negative for other markers, CD45, CD11b, and CD34 after days 1, 4, and 7 of culture (Fig. 2). The flow cytometry analysis was confirmed by gene expression analysis which revealed that ADSCs encapsulated within all the AM hydrogels showed higher expression of SOX-2 and OCT-4 compared to the TCP group. Both SOX-2 and OCT-4 expression were significantly higher in AM8 compared to TCP (Fig. 3). SOX-2 and OCT-4 were found to be expressed in both murine and human ADSCs and are associated with cell self-renewal and pluripotency. The engraftment of ADSCs in the AM hydrogel resulted in increased SOX-2 and OCT-4 levels, indicating that the AM hydrogel maintained ADSC stemness, thereby, functioning as a stem cell niche. Moreover, the AM hydrogel may also synergize the paracrine effect of ADSCs by providing an instructive substrate to guide ADSC proliferation and paracrine factor production, potentially increasing the therapeutic effect of ADSCs.


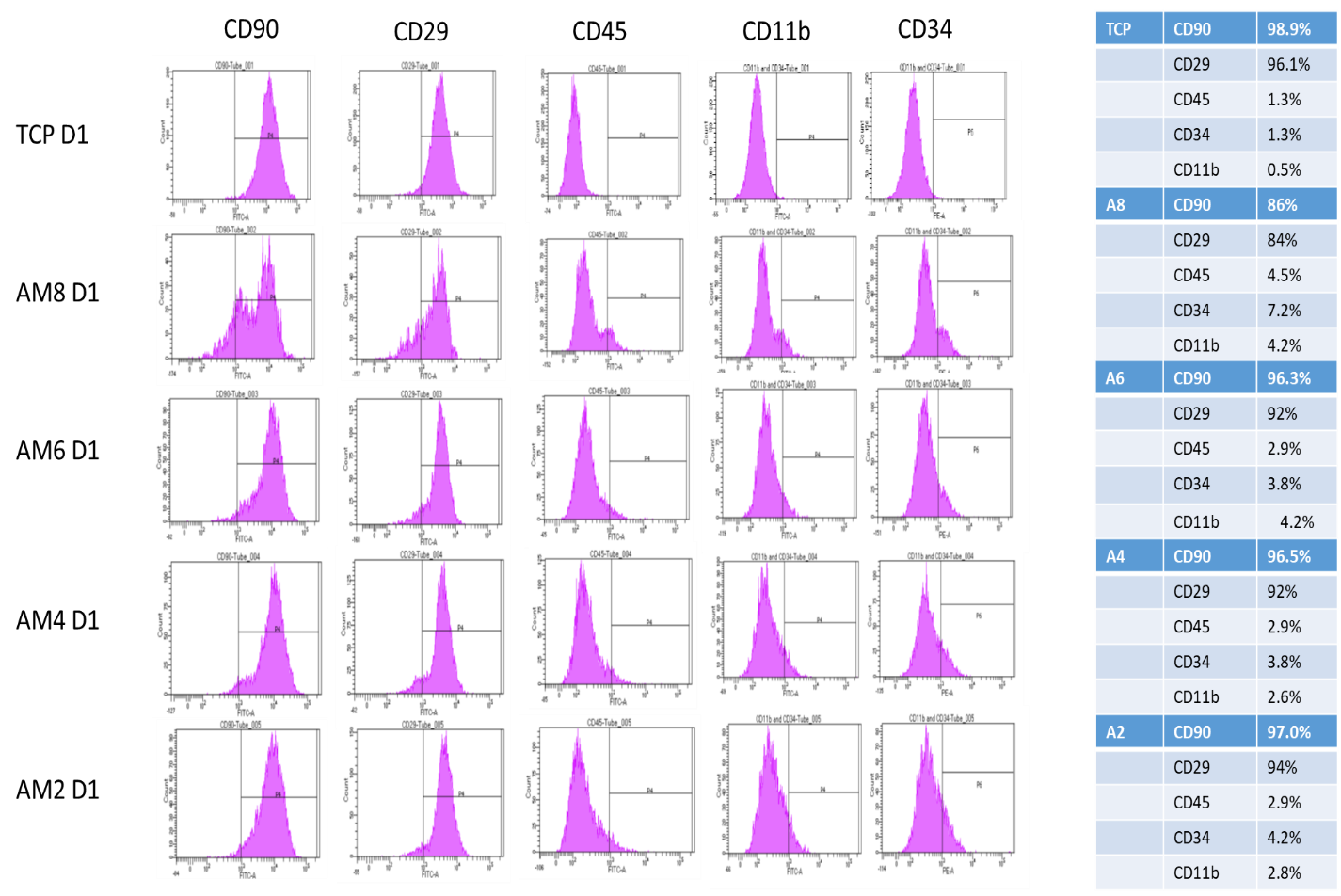


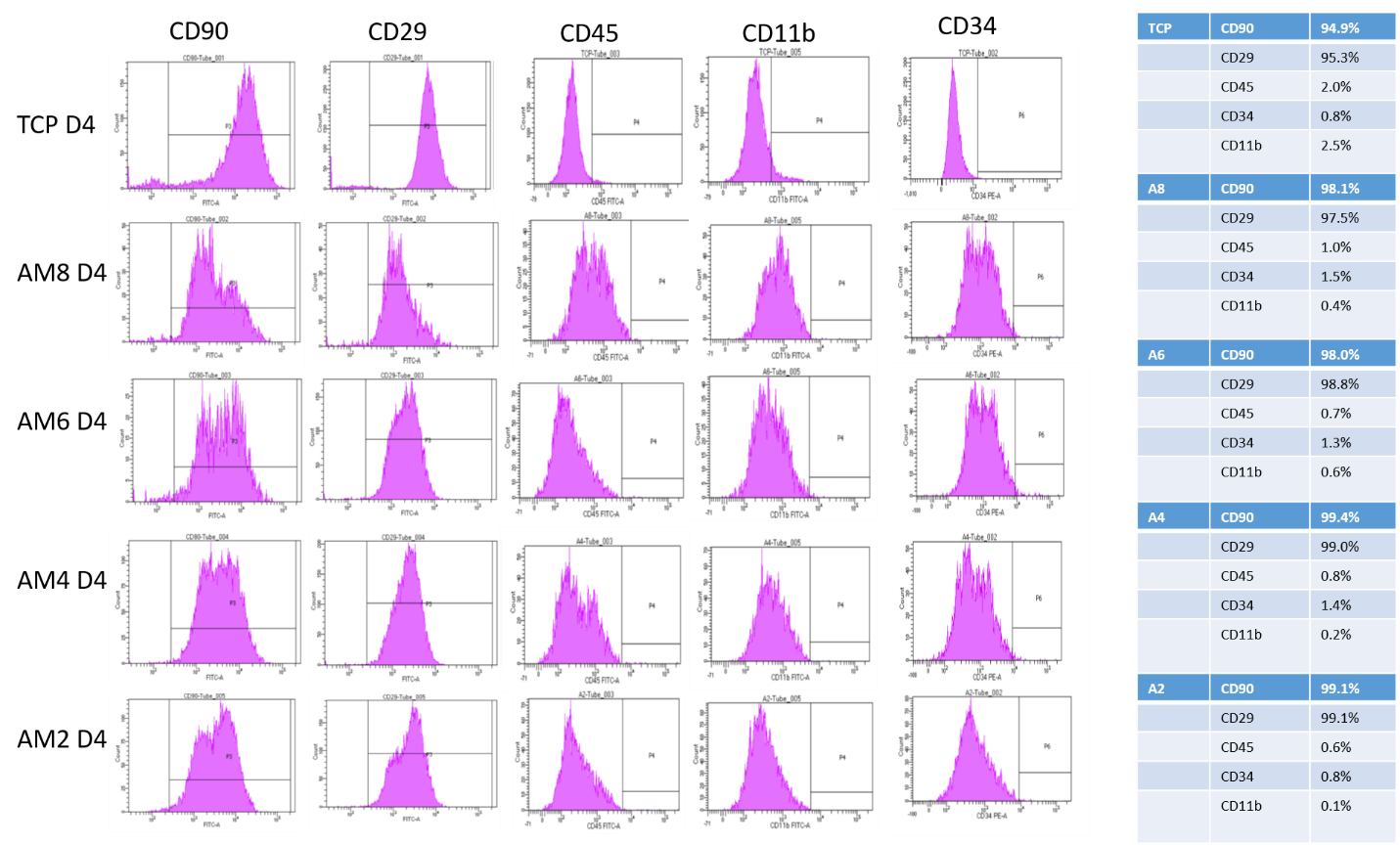


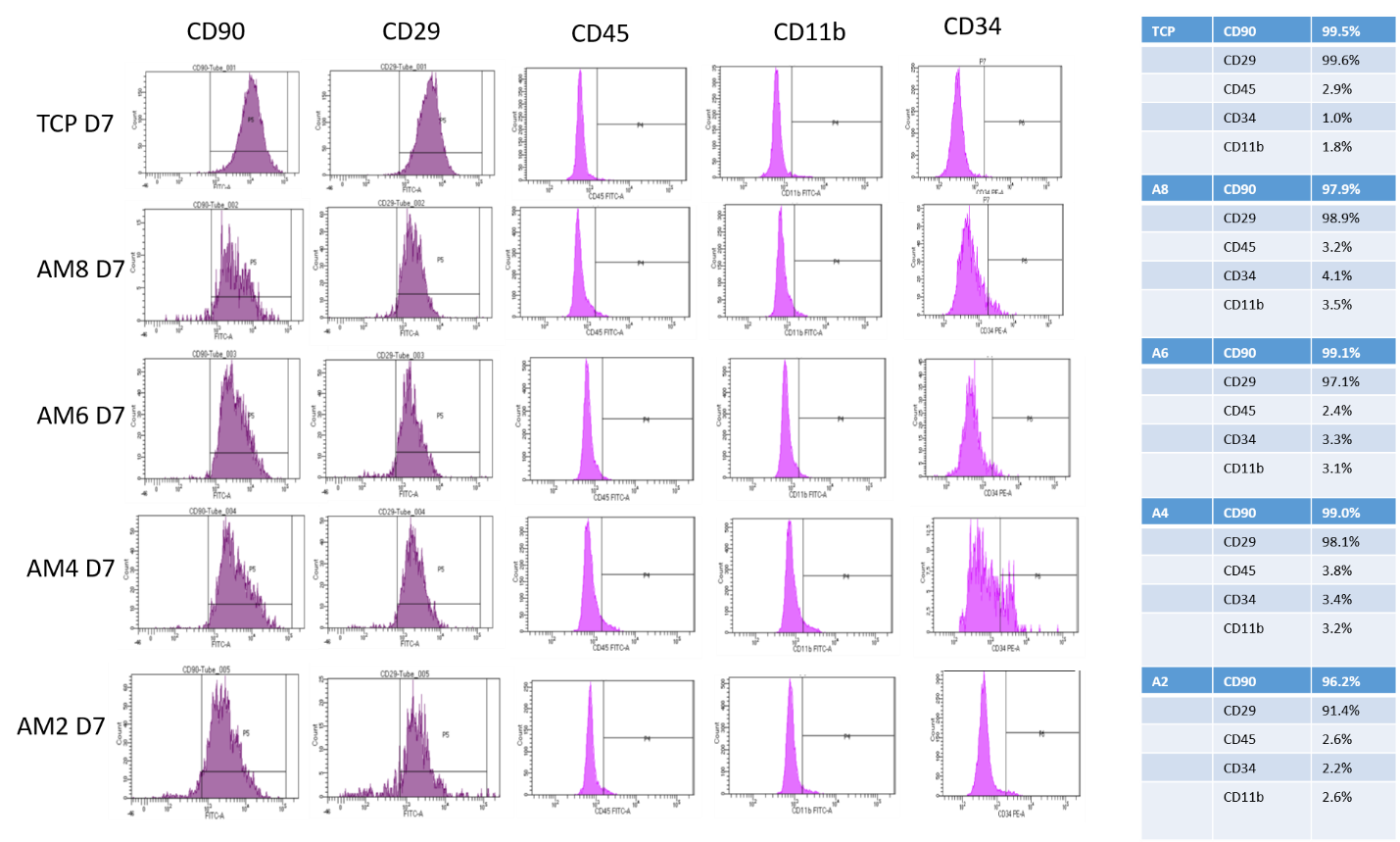


Fig S2. Flow cytometry showing stemness of ADSCs within AM gels at 1, 4, 7 days. n=2


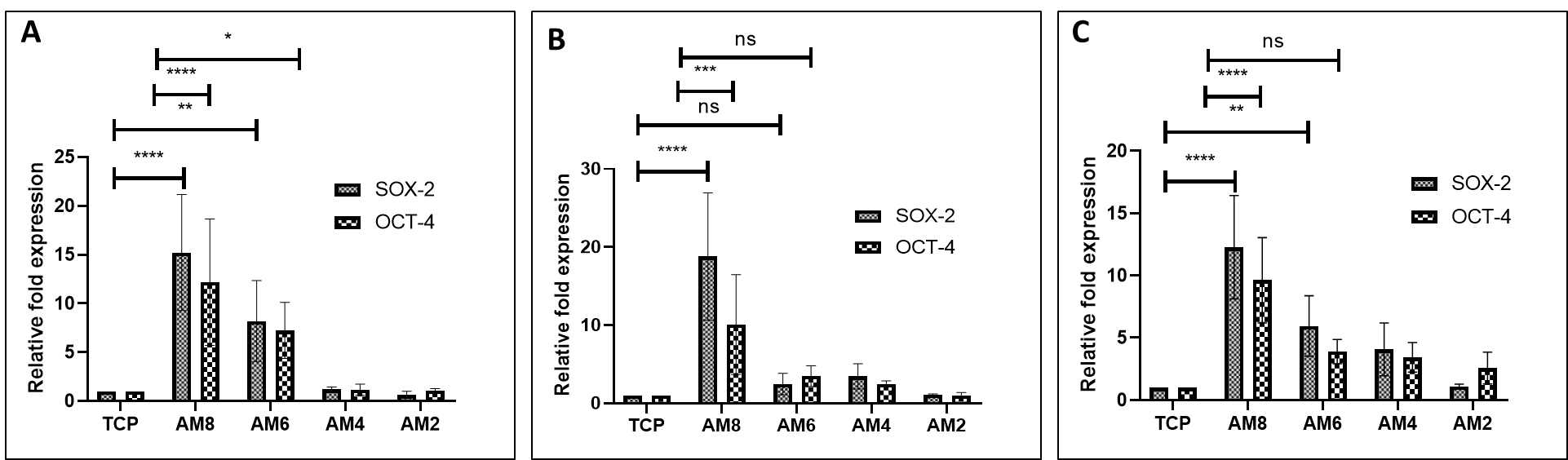


Fig. S3. Gene expression showing ADSC stemness maintained within the hydrogels after (A) day 1, (B) day 4, (C) day 7, mean and SD with n = 3, repeated twice, [****p < 0.0001, ***p < 0.001, **p < 0.01, *p < 0.05].
